# The interplay between eosinophils and T cells in breast cancer immunotherapy

**DOI:** 10.1002/1878-0261.13413

**Published:** 2023-03-17

**Authors:** Ioannis Zerdes, Alexios Matikas, Theodoros Foukakis

**Affiliations:** ^1^ Breast Center, Theme Cancer Karolinska University Hospital and Karolinska Comprehensive Cancer Center Stockholm Sweden; ^2^ Department of Oncology/Pathology Karolinska Institutet Stockholm Sweden

**Keywords:** breast cancer, eosinophils, immunotherapy, T cells, tumor microenvironment

## Abstract

Treatment with immune checkpoint inhibitors (ICI) has revolutionized cancer management for multiple tumor types, including breast cancer. However, not all patients respond to ICI, and unraveling the determinants and mechanisms of response still remains an unmet need. A recent study has uncovered the critical role of eosinophils in mediating immunotherapy effect in breast cancer, mainly by stimulating the activation of CD8+ T‐cells. Furthermore, the intratumoral eosinophil recruitment was directed by CD4+ T cells and the interleukins IL‐5 and IL‐33, thus providing the rationale for targeting eosinophils to enhance ICI response.

## Graphical abstract

Immune checkpoint inhibitors (ICI) have revolutionized the therapeutic management of multiple tumor types, yet not all patients respond to ICI. A recent study demonstrated the critical role of eosinophils in mediating immunotherapy effect in breast cancer, mainly by stimulating CD8+ T‐cell activation. Intratumoral eosinophil recruitment was directed by CD4+ T‐cells, IL‐5 and IL‐33, thus providing the rationale for targeting eosinophils to enhance ICI response.

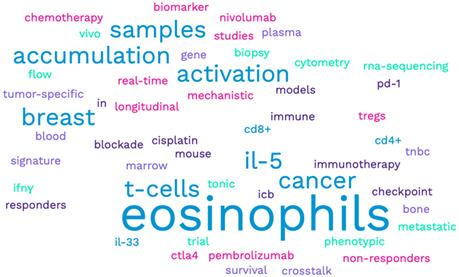

**Abbreviations**
CTLA4: cytotoxic T‐lymphocyte‐associated protein 4
ICI: immune checkpoint inhibitors
KEP: keratin14‐cre;Cdh1^F/F^;Trp53^F/F^
PD1: programmed death protein 1
TME: tumor microenvironment
TNBC: triple‐negative breast cancer

Immune checkpoint inhibitors (ICI) have revolutionized the management of multiple cancer types, among others triple‐negative breast cancer (TNBC) [1, 2, 3]. However, not all patients respond to ICI, and there is thus a need for reliable biomarkers beyond PD‐L1 protein expression – which in itself is an imperfect predictor of benefit – such as tissue‐based tumor microenvironment (TME) and circulating markers, genomic alterations and gene expression signatures [4]. Previous studies have investigated the molecular determinants and genomic features of response in breast cancer patients treated with ICI, mostly in metastatic TNBC [5, 6]. Recent single‐cell sequencing and multi‐omics studies have demonstrated the critical role of T cells, macrophages and dendritic cells as well as their dynamic alteration in ICI responders [7, 8, 9], thus highlighting the potential interplay among the innate and adaptive immunity components.

In a study published in *Cancer Cell* in January 2023 [10], Blomberg et al. uncovered a mechanism in which upon stimulation by CD4+ T cells as well as IL‐5 and IL‐33 factors, eosinophils could enhance CD8+ T‐cell activation and ICI effect. By using flow cytometry in pre‐ and on‐treatment blood samples from TNBC patients treated with nivolumab [11], the researchers observed a systemic accumulation of eosinophils in responders. This increase was even associated with improved progression‐free and overall survival. In addition, an intratumoral enrichment of eosinophil‐related gene expression was noted in responders from the same patient cohort and correlated also with CD8+ T‐cell gene signatures, providing an indication for potential crosstalk. These findings were confirmed in spontaneous primary and metastatic *Keratin14‐cre;Cdh1* ^*F/F*^ *;Trp53* ^*F/F*^ (KEP) mouse models which showed increased survival and durable responses, respectively, upon combinational treatment with cisplatin and anti‐PD1 + anti‐CTLA4 (CIS + ICI). This response was accompanied by increased T‐cell abundance and respective activation markers and cytokines as well as eosinophils with enriched IFN‐γ response. Importantly, the treatment outcome was dependent on eosinophils, CD8+ T cells and their interplay since eosinophil depletion abrogated CD8 T‐cell activation and the synergistic CIS + ICI effect. Moreover, eosinophils facilitated their action through the activation of CD8+ T cells, which were in turn recruited intratumorally upon CIS + ICI treatment.

To further elucidate the underlying mechanism of eosinophil accumulation, the authors proposed that CD4+ T cells produced IL‐5 which stimulated the production of eosinophils in bone marrow *in vivo*. This ICI‐induced IL‐5 production was also tested *ex vivo* using a patient‐derived tumor fragment platform for treatment with ICI. The CD4+ T‐cell origin of IL‐5 was confirmed at the patient level, where *IL‐5* mRNA was increased in isolated CD4+ T cells in on‐nivolumab samples. Next, the authors wanted to investigate potential factors that could induce the intratumoral eosinophil recruitment. Towards this end, broad cytokine/chemokine profiling revealed increased levels of IL‐33, both in *in vivo* mouse models and in TNBC patients treated with ICI, which was also correlated with eosinophil gene signature in responders. Of note, IL‐33 blockade abrogated CD8+ T‐cell activation and the intratumoral but not the systemic eosinophilic accumulation and abolished the CIS + ICI treatment effect. In contrast, treatment of tumor‐bearing mice with recombinant IL‐33 and ICI *in vivo* led to induction of eosinophils and subsequent activation of CD8+ T cells and resulted in improved tumor control and survival prolongation.

Taken together, these findings revealed the functional role of eosinophils which could serve as mediators of the synergistic chemoimmunotherapy effect in breast cancer. Given that eosinophils are bone‐marrow‐derived granulocytes involved mainly in parasitic infections, autoimmune diseases and allergic asthma [12], a growing body of literature recognizes their association with antitumor immunity and immunotherapy response [13]. Indeed, the authors of this study have also demonstrated an eosinophilic enrichment in ICI responders with other tumor types, including advanced non‐small cell lung cancer and early‐stage mismatch repair‐proficient colon cancer. However, their role in breast cancer immunotherapy is largely unexplored and the underlying mechanisms require further investigation [14]. Furthermore, following the asthma and inflammatory diseases paradigm, the impact of the CD4+ T‐cells ‐IL‐5 – IL‐33 axis on the activation, accumulation and migration of eosinophils could share common pathophysiology features with cancer. Importantly, in light of this study's results, IL‐33 could represent an appealing treatment target in order to boost the effect of immunotherapy and possibly that of other therapies in breast cancer. The potential of inducing the expression of the tumor‐derived alarmin IL‐33 and consequently eosinophil accumulation has also been previously described, using the dipeptidyl peptidase DPP4 (CD26) inhibitor sitagliptin in multiple solid tumors *in vivo*, including breast cancer models [15]. Nonetheless, the establishment of eosinophil‐based treatment strategies needs to be prospectively tested in clinical trials.

Despite the important findings of this study, there are several key points which warrant further discussion. First, cisplatin and anti‐CTLA4 have not been used as treatment regimens in the pivotal breast cancer immunotherapy trials. Given the conflicting results on the optimal chemotherapy partner for TNBC immunotherapy [2, 16], testing the role of eosinophils upon (nab)paclitaxel‐including chemoimmunotherapy combinations would be of relevance. Second, is this effect confined only in TNBC or even in hormone receptor‐positive disease? Third, is the presence of eosinophils only a predictive biomarker of ICI response as previously described [13] or could it provide some therapeutic insights? Regarding the former, its validity and utility as a real‐time biomarker need to be investigated in larger immunotherapy‐treated patient cohorts. Moreover, given that the effector functions of eosinophils have been linked to enhanced potency of various treatment modalities including CAR T‐cells, their impact on different treatment combinations (with or without chemotherapy) in other tumor types remains an open question. Last but not least, how eosinophils activate CD8+ T cells and the exact origin of IL‐33 remain to be addressed in order to increase our understanding of the mechanistic background behind their synergy.

In conclusion, this study uncovered a critical interplay between eosinophils and T cells, thus highlighting the role of TME components in the regulation of anti‐tumor immunity and ICI response. Whether eosinophil accumulation could be used only as a biomarker of treatment efficacy and/or lead to the development of new therapeutic avenues in breast and other tumors needs to be further elucidated.

## Conflict of interest

Ioannis Zerdes has no conflict of interest to declare. Alexios Matikas: consultancy to Veracyte (no financial or other compensation) and Roche (no financial or other compensation). Theodoros Foukakis: consultancy to Astra Zeneca, Affibody, Pfizer, Novartis, Veracyte, Exact Sciences, Gilead Sciences and Roche; honoraria from UpToDate; research funding to institution from Pfizer, Astra Zeneca, and Novartis.
